# Activation of NRF2/FPN1 pathway attenuates myocardial ischemia–reperfusion injury in diabetic rats by regulating iron homeostasis and ferroptosis

**DOI:** 10.1007/s12192-022-01257-1

**Published:** 2022-02-05

**Authors:** Hao Tian, Yonghong Xiong, Yi Zhang, Yan Leng, Jie Tao, Lu Li, Zhen Qiu, Zhongyuan Xia

**Affiliations:** 1 Department of Anesthesiology, Renmin Hospital of Wuhan University, Hubei Province, Wuhan, 430060 China; 2 Department of Anesthesiology and Perioperative Medicine, Shandong Qianfoshan Hospital, Cheeloo College of Medicine, Shandong University, Jinan, Shandong China

**Keywords:** NRF2, FPN1, Iron homeostasis, Ferroptosis, Diabetes, Myocardial ischemia–reperfusion injury

## Abstract

In patients with ischemic heart disease, myocardial ischemia–reperfusion injury (IRI) can aggravate their condition even worse, and diabetes increases their risk of myocardial IRI. Pathological pathways of common diseases and surgical operations like diabetes, obesity, coronary artery angioplasty, and heart transplantation entail disorders of iron metabolism. Ferroportin1 (FPN1) is the only mammalian protein associated with iron release and thus plays a vital role in iron homeostasis, while nuclear factor E2-related factor 2 (NRF2) controls the transcription of FPN1. Since the NRF2/FPN1 pathway may play a favorable role in the therapy of diabetic myocardial IRI, this work investigated the possible mechanism. In this study, we investigated the effects of ferroptosis in STZ-induced diabetic rats following myocardial IRI in vivo, and its alteration in glucose and hypoxia/reoxygenation-induced cardiomyocytes injury in vitro. Rats and H9c2 cardiomyocytes were randomly divided into 6 groups and treated with sulforaphane and erastin besides the establishment of diabetic myocardial IRI and hyperglycemic hypoxia-reoxygenation models. Cardiac functional and structural damage were detected by Evans blue/TTC double staining, echocardiography, HE staining, and serological indices. CCK-8 assay and ROS production were used to measure cardiomyocyte viability and oxidative stress level. Additionally, the changes in cell supernatant levels of Fe^2+^, SOD, MDA, and mRNA and protein expression of ferroptosis marker proteins confirmed the beneficial effects of the NRF2/FPN1 pathway on diabetic myocardial IRI related to iron metabolism and ferroptosis. Overall, these findings suggest that iron homeostasis-related ferroptosis plays an important role in aggravating myocardial IRI in diabetic rats, and NRF2/FPN1 pathway-mediated iron homeostasis and ferroptosis might be a promising therapeutic target against myocardial IRI in diabetes.

## Introduction

Diabetes is a chronic metabolic disease marked by high blood sugar levels, and its prevalence and disability rates have risen dramatically in recent years. Persistent hyperglycemia, especially in the heart and blood vessels, can induce chronic damage and dysfunction of many tissues. Diabetic people are at risk of developing ischemic heart disease, particularly acute myocardial infarction. Coronary blood flow must be restored in order to rescue the ischemic myocardial (Crisafulli et al. 2020). However, ischemia–reperfusion injury (IRI) can occur during this process, resulting in worsened structural and functional damage (Davidson et al. 2019). Diabetes, according to prior study, can worsen myocardial IRI by causing oxidative stress via associated pathways that trigger apoptosis, inflammation, autophagy, and so on (Gan et al. 2020; Qu et al. 2019; Wang et al. 2017). However, the main related mechanisms of myocardial IRI in diabetes need to be further clarified.

Ferroptosis is a recently recognized form of iron-dependent programmed cell death that based on the inactivation of the lipid repair enzyme glutathione peroxidase 4 (GPX4) and subsequent accumulation of reactive oxygen species (ROS) in lipids. Iron chelators, such as desferrioxamine, have been demonstrated in studies to greatly reduce erastin-induced cellular ferroptosis. This indicates that iron homeostasis is crucial in the development of cellular ferroptosis (Dixon et al. 2012). Other studies have found that polyunsaturated fatty acid-containing cell and plasma membranes are particularly vulnerable to lipid radical peroxidation, and that the pace of this reaction is considerably accelerated in the presence of iron (Galluzzi et al. 2012). Thus, irons are required for the accumulation of lipid peroxide and the commencement of the ferroptosis process.

Iron is an indispensable trace metal element in a variety of physiological activities. Both excess and deficiency of iron are potential risk factors. Imbalances in iron homeostasis have been reported to be involved in a variety of diseases such as cancer, anemia, neurodegenerative diseases, and heart disease (Gozzelino and Arosio 2016). Iron uptake and iron release are key steps in the regulation of cellular iron metabolism. It is well known that the occurrence of disorders of iron metabolism in the body is associated with abnormalities of hepcidin. And ferroportin1 (FPN1) internalization and degradation mediated by hepcidin are essential in the maintenance of cardiac iron homeostasis (Lakhal-Littleton et al. 2015). Multiple iron input mechanisms exist in cardiomyocytes. But FPN1 is the only iron release-related protein found in mammals and plays a critical role in systemic iron homeostasis. FPN1 is a transmembrane protein that is responsible for iron export from the cell to the plasma and is encoded by the SLC40A1 gene (Lakhal-Littleton et al. 2015).

Nuclear factor E2-related factor 2 (NRF2), known as a critical regulator of the antioxidant response, is a transcription factor encoded by the NFE2L2 gene and belongs to the leucine zipper structure family (Moi et al. 1994). Oxidative stress plays an important role in the process of diabetes and its complications (Brownlee 2005). And oxidative stress is considered as one of the main causes of cardiac dysfunction during myocardial ischemia–reperfusion (Ichihara 2013). Zhang et al. have demonstrated that myocardial oxidative damage and cell death could be ameliorated by activating NRF2-associated signaling pathways, and thus the myocardial IRI of T1D was alleviated (Zhang et al. 2018). And in the nucleus, NRF2 acts as a transcription factor to regulate iron metabolism-related gene SLC40A1 (Harada et al. 2011). Of note, iron regulatory proteins (IRPs) can recognize and bind iron responsive elements (IREs) present in the 5′-untranslated or in the 3′-untranslated regions of the mRNAs encoding proteins involved in iron metabolism (Iwai 2019). Therefore, the regulation of iron homeostasis is not limited to the transcriptional level but can also be obtained through post-transcriptional regulation. Research has reported that the expression of FPN1 was decreased in NRF2 knockout mice (Liu et al. 2020a). Furthermore, studies have found that myocardium could be protected from ferroptosis by regulating NRF2 expression (La Rosa et al. 2021; Luo et al. 2021). However, the NRF2-FPN1 interaction relationship in diabetic myocardial IRI has not been reported. Therefore, the purpose of our present study aims to test the hypotheses that NRF2/FPN1 signaling pathway-mediated iron homeostasis and ferroptosis play a crucial role in diabetic myocardial IRI, which may be a protective mechanism against diabetic myocardial IRI.

## Materials and methods

### Animals and diabetes induction

The animal experimental protocol was approved by the Bioethics Committee of Renmin Hospital of Wuhan University (Animal Welfare No.20200303). Healthy male adult Sprague–Dawley (SD) rats were supplied by Beijing Vital River Bioscience Co. Ltd, weighing 200–220 g, 6–8 weeks old. After 5 days of acclimatization, the rats were fasted for 12 h for diabetes induction. The diabetic rats were administered a single intraperitoneal injection of 60 mg/kg streptozotocin (STZ) dissolved in citrate buffer to induce diabetes as described previously (Qiu et al. 2017). The non-diabetic rats were injected with an equal volume of sodium citrate buffer. After 72 h (with 6 h fasting), the rats exhibiting hyperglycemia (blood glucose level higher than 16.7 mmol/l), polyphagia, polydipsia, and polyuria were considered to be suffering from diabetes.

### Reagents

Streptozotocin (STZ), triphenyl tetrazolium chloride (TTC), and Evans blue (EB) were purchased from Sigma Chemical Co. (MO, USA). Dulbecco’s modified Eagle’s medium (DMEM) and fetal bovine serum (FBS) were obtained from Gibco Laboratories (Grand Island, NY, USA). Sulforaphane (SFN), the NRF2 activator, was purchased from Glpbio Co. (CA, USA). Erastin (Era), the ferroptosis inducer, was purchased from Selleck (Houston, TX). The cell counting kit-8 (CCK-8) and 2′,7′-dichlorofluorescein diacetate (DCFH-DA) assay test kits were obtained from Beyotime Institute of Biotechnology (Shanghai, China). Lactate dehydrogenase (LDH), creatine kinase-MB (CK-MB), superoxide dismutase (SOD), and malondialdehyde (MDA) assay test kits were purchased from Nanjing Jiancheng Bioengineering Institute (Nanjing, Jiangsu, China). Fe^2+^ assay test kit was purchased from Abcam Co. (Abcam, UK). The primers for NRF2, FPN1, ACSL4, and β-actin were designed and synthesized by Wuhan Servicebio Co. (Wuhan, Hubei, China). NRF2 primary antibodies were purchased from Cell Signaling Technology (CST, Beverly, CA, USA). FPN1, ACSL4, GPX4, and GAPDH primary antibodies were obtained from Proteintech Co. (Wuhan, Hubei, China).

### Glucose tolerance testing

The diabetic rats were fasted overnight, and then glucose was administered at a dose of 2 g/kg by gastric lavage or intraperitoneally injection to conduct the oral glucose tolerance test (OGTT) and intraperitoneal glucose tolerance test (IPGTT) to confirm the success of animal model. Blood glucose level was measured at 0 (before glucose load), 30, 60, 90, and 120 min. All blood samples were collected from the caudal vein to determine plasma glucose.

### Myocardial ischemia and reperfusion model

Rats were anesthetized by intraperitoneal injection of 1% sodium pentobarbital (60 mg/kg) and managed for electrocardiogram (ECG) monitoring. After disinfection of skin, tracheotomy was performed and the ventilator was connected for mechanical ventilation. The ribs were cut at the 3rd to 4th intercostal space in the left midclavicular line and the entire heart was fully exposed. Subsequently, the left anterior descending coronary artery (LAD) below the left auricle was ligated with a 7–0 silk wire. Successful ischemia was indicated when there was a change in color from red to white in the apical region and left ventricular wall with reduced ventricular wall motion and an elevated ST-segment arch in the ECG. After 30 min, the apical and left ventricular color gradually returned to red and the ST-segment recovered, suggesting successful reperfusion, as seen by loosening the ligature. Lastly, the myocardium was reperfused for 2 h (for protein expression and serological indicators measurement) or 72 h (for cardiac function measurement). Furthermore, the ligation thread was passed through the LAD but not ligated in the sham operation group.

### Experimental protocols

For the in vivo study, 8 weeks after STZ injection, all rats were randomly divided into 4 groups: (1) normal + sham group (NS); (2) normal + ischemia–reperfusion group (NIR); (3) diabetes + sham group (DS); (4) diabetes + ischemia–reperfusion group (DIR). To gain a deeper insight into the effect of NRF2 activation, the following experiments were preformed among diabetic rats: (1) diabetes + ischemia–reperfusion group (DIR); (2) diabetes + ischemia–reperfusion + sulforaphane group (DIR + SFN); (3) diabetes + ischemia–reperfusion + SFN + erastin group (DIR + SFN + Era). Each group contains 8 rats. The NRF2 activator sulforaphane (500 μg/kg/day) was injected intraperitoneally for 3 days before ischemia (Piao et al. 2010). And the ferroptosis inducer Erastin (20 mg/kg) was injected intraperitoneally at the beginning of the ischemia–reperfusion operation.

**Fig. 1:**
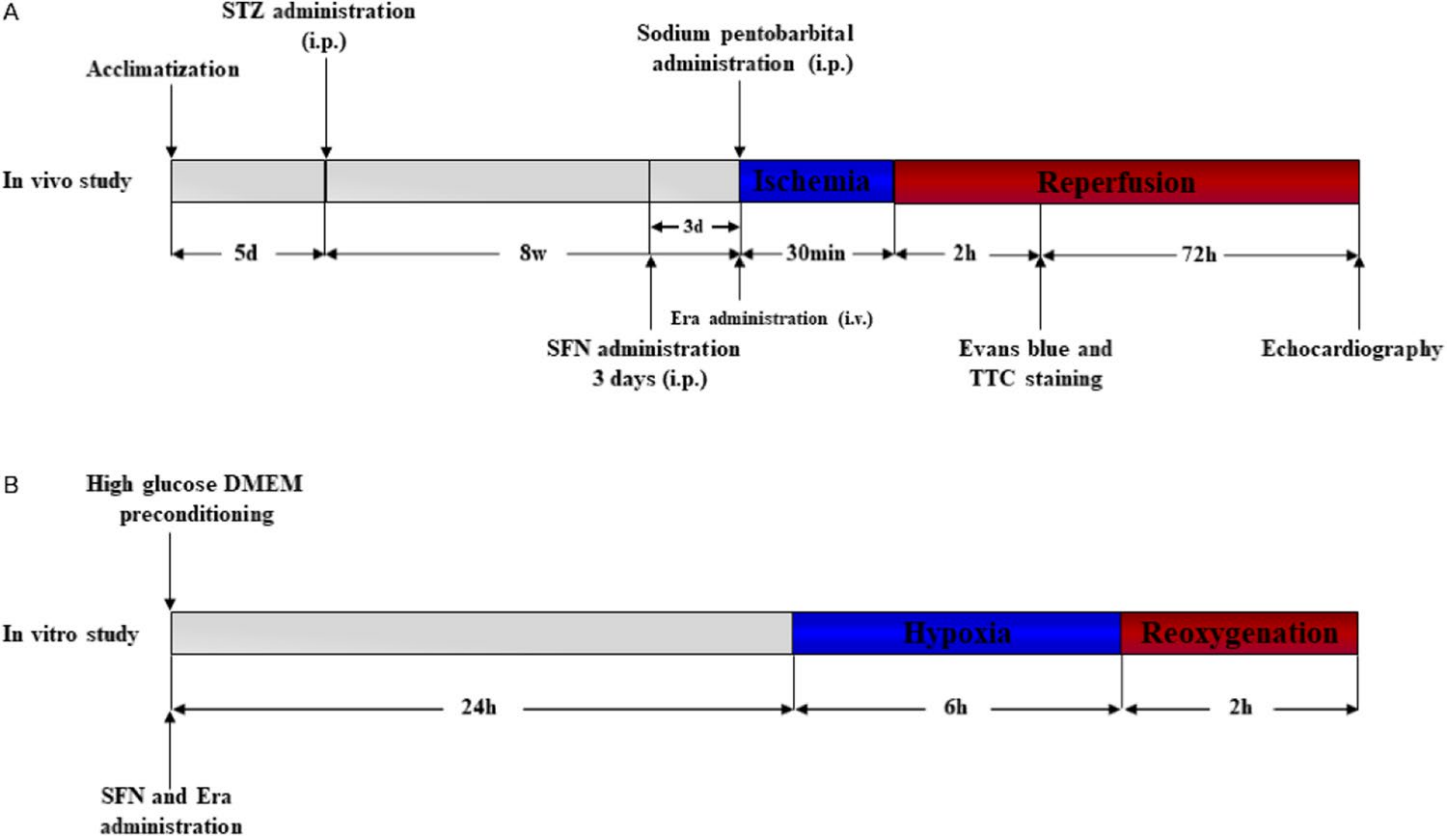
Experimental protocols. Illustration showing the experimental procedures including diabetes induction, ischemia–reperfusion model, SFN and Era administration, and sacrifice end point. **A** In vivo experimental protocol. **B** In vitro experimental protocol. *STZ* streptozotocin, *SFN* sulforaphane, *Era* erastin

For the in vitro study, H9c2 cardiomyocytes, a subclonal cell line derived from embryonic BD1X rat myocardial tissue, were randomly assigned to 6 groups: (1) normal glucose group (5.5 mM); (2) normal glucose + hypoxia-reoxygenation group (H/R); (3) high glucose group (HG) (30 mM); (4) high glucose + hypoxia-reoxygenation group (HH/R); (5) high glucose + hypoxia-reoxygenation + sulforaphane group (HH/R + SFN); (6) high glucose + hypoxia-reoxygenation + sulforaphane + erastin group (HH/R + SFN + Era). H9c2 cardiomyocytes of all groups were cultured in low-glucose DMEM containing 10% FBS and 1% penicillin/streptomycin and incubated in normoxic incubator at 37 ℃ in a humidified atmosphere of 5% CO_2_. When the density of H9c2 cardiomyocytes reached 70–80%, all cells were digested with trypsin containing ethylenediaminetetraacetic acid (EDTA) and transferred to 6-well culture plates for subsequent experimental treatments. To simulate the high glycemic state, cells were cultured in serum-free medium overnight and exposed to HG medium for 24 h. For the H/R cell model, cells were subjected to hypoxic state (94% N_2_ + 5% CO_2_ + 1% O_2_) for 6 h and followed by reoxygenation (95% air + 5% CO_2_) for 2 h. Sulforaphane and erastin was given 24 h before H/R. The experimental protocol is depicted schematically in Fig. 1.

### Determination of myocardial infarction

After 2 h of ischemia–reperfusion treatment, six rats in each group were randomly selected to ligate LAD along with the original site. The 2% Evans Blue reagent was slowly injected from the femoral vein, while the non-ischemic area showed blue staining region. The aorta was immediately clamped and the heart was removed and sliced along the longitudinal axis. Five pieces of heart slices were prepared, placed in the 1% TTC solution in a 37 ℃ incubator for 30 min in the dark. The slices were then fixed in 4% paraformaldehyde for 30 min, scanned by the scanner (Epson, v30, Japan). And the myocardium area was calculated with an image analysis system (Image J; National Institutes of Health, USA). Theoretically, normal myocardium appears blue, ischemic myocardium shows brick-red, and infarcted myocardium displays pale. For ischemic myocardium stained by TTC staining, brick-red myocardium was defined as the area at risk (AAR) and pale myocardium was defined as the infarct area (IA). The percentage of myocardial infarction area was calculated as IA versus AAR (IA/AAR × 100%).

### Echocardiography

In order to avoid the interference of residual gas in the thoracic cavity on the echocardiography after thoracotomy, 6 rats were taken from each group after 72 h of reperfusion. Rats were anesthetized with 2–3% inhaled isoflurane and fixed on the operating table. The MyLab 30CV ultrasound system (Biosound Esaote, Indianapolis, IN, USA) with a 15-MHz transducer probe was used to perform two-dimensional and M-mode echocardiographic measurements. Parasternal long axis and short axis images were obtained in short and long axes in two-dimensional and M-mode for quantification. Left ventricular internal dimension systole (LVIDs) and left ventricular internal dimension diastole (LVIDd) were measured on the parasternal LV long axis view. Left ventricular ejection fraction (LVEF) and left ventricular shortening fraction (LVFS) were calculated by computer algorithms. All measurements that represented the mean of 5 consecutive cardiac cycles were performed in a blinded manner.

### Hematoxylin and eosin staining

Myocardial tissue was fixed overnight in 4% paraformaldehyde at 4 °C. The tissue was then dehydrated in a series of ethanol and xylene solutions and embedded in paraffin wax. A microtome was used to slice the heart tissue into slices of 4 μm thick. Hematoxylin and eosin (HE) staining was used to examine the morphological changes and damage severity as instructed by the manufacturer.

### Measurement of serum CK-MB and LDH levels

At the end of reperfusion, arterial blood samples were collected at the apex of the heart and centrifuged at 1200 rpm for 15 min. The creatine kinase-MB (CK-MB) was detected by the assay kit and lactate dehydrogenase (LDH) was detected by colorimetry according to the manufacturer’s instructions.

### Cell viability assay

Cells were plated in 96-well plates at a density of 3000 cells/well and grouped according to the experiment (5 replicate wells per group). After cells were cultured and treated in 96-well plates, 10 μL of CCK-8 reagent was added to each well and then incubated for 2 h in the dark. Absorbance was detected at 450 nm with a microplate reader (Perkin Elmer, USA).

### *MDA, SOD, and Fe*^*2*+^*determination*

After modeling, the cells and cell supernatant were collected to measure the MDA level, antioxidant enzyme SOD activity and intracellular Fe^2+^ concentration by spectrophotometry according to the manufacturer’s instructions. Then, the intensity was observed by a microplate reader.

### ROS measurement

Intracellular reactive oxygen levels were measured with the 2′,7′-dichlorofluorescein diacetate (DCFH-DA) molecular probe. Briefly, cardiomyocytes in 6-well plates were loaded with the appropriate amount of DCFH-DA probe for 30 min at 37 °C in the dark. DCFH-DA was oxidized and converted to highly fluorescent DCFH and showed green fluorescence in the cytoplasmic lysate. Fluorescence images were captured by a fluorescence microscope (Olympus, Tokyo, Japan).

### Immunofluorescence

H9c2 cardiomyocytes in 6-well plates were fixed with 4% paraformaldehyde for 30 min. After that, they were permeabilized in 0.5% Triton X-100 at 37 °C for 20 min, blocked with 5% BSA solution and add NRF2 (1:100, ABclonal, Wuhan, Hubei, China) antibody to incubate overnight at 4 °C. Finally, the samples were incubated with Cy3 labeled goat anti-rabbit secondary antibody, followed by 4′,6-diamidino-2-phenylindole dihydrochloride (DAPI) (Invitrogen, Carlsbad, CA, USA) staining for 10 min, then observed and captured positive areas with a laser confocal microscope (Leica TCS, Germany).

### Quantitative real-time polymerase chain reaction (qRT-PCR) analysis

Total RNA was extracted from the myocardial samples and H9c2 cells using Trizol reagent. Two micrograms of RNA from each sample was then reverse transcribed into cDNA according to the Prime-Script RT reagent kit instruction (Servicebio, Wuhan, Hubei, China). The qRT-PCR was performed using a SYBR Green qPCR Reagent Kit (Servicebio, Wuhan, Hubei, China) by Bio-Rad CFX Connect Real-Time PCR Detection System (Bio-Rad, USA). The used primers are listed in Table 1. The mRNA levels were normalized to β-actin mRNA level. The expression of genes was analyzed by using the 2^−ΔΔCT^ method.

**Table 1:** The sequences of RT-PCR used in this study

Gene	Forward (5′-3′)	Reverse (5′-3′)
ACSL4	CTGCCGAGTGAATAACTTTGGA	TCAGATAGGAAGCCTCAGACTCATT
NRF2	TTGGGGTAAGTCGAGAAGTGTTT	ATGTGGGCAACCTGGGAGTA
SLC40A1	CTAAATCCGTCCCCATAATCTCC	CCCATTGCCACAAAGGAGAC
β-actin	TGCTATGTTGCCCTAGACTTCG	GTTGGCATAGAGGTCTTTACGG

### Western blotting

H9c2 cardiomyocytes and cardiac tissue were homogenized in pre-cooled RIPA lysis buffer following various treatments. The supernatant was taken and added to the protein buffer. Then, the extracts were placed at 100 °C and keep them for 5 min. Fifty micrograms of total protein per well was separated by SDS-PAGE gel and then transferred to PVDF membranes. For 1 h, the membranes were incubated in 5% skimmed milk. Primary antibodies (ACSL4, 1:1000; GPX4, 1:1000; NRF2, 1:1000; FPN1, 1:1000; GAPDH, 1:1000) were incubated with the membranes overnight at 4 °C. Secondary antibody (1:5000, Proteintech) was incubated for 1 h at room temperature with horseradish peroxidase (HRP) conjugated secondary antibody. The biological image analysis system was used for the final analysis (Bio-Rad, USA). Each sample’s GAPDH levels were used to determine the relative expression levels of the target proteins.

### Statistical analysis

All results were analyzed using Graphpad Prism 8.0 software (GraphPad Software, USA) and presented as means ± standard deviation. Statistical analysis was carried out using one-way analysis of variance (ANOVA) and *P* < 0.05 was deemed significant.

## Results

### The exacerbated myocardial IRI in diabetic rats was associated with the activation of ferroptosis and the downregulation of the NRF2/FPN1 pathway

**Fig. 2:**
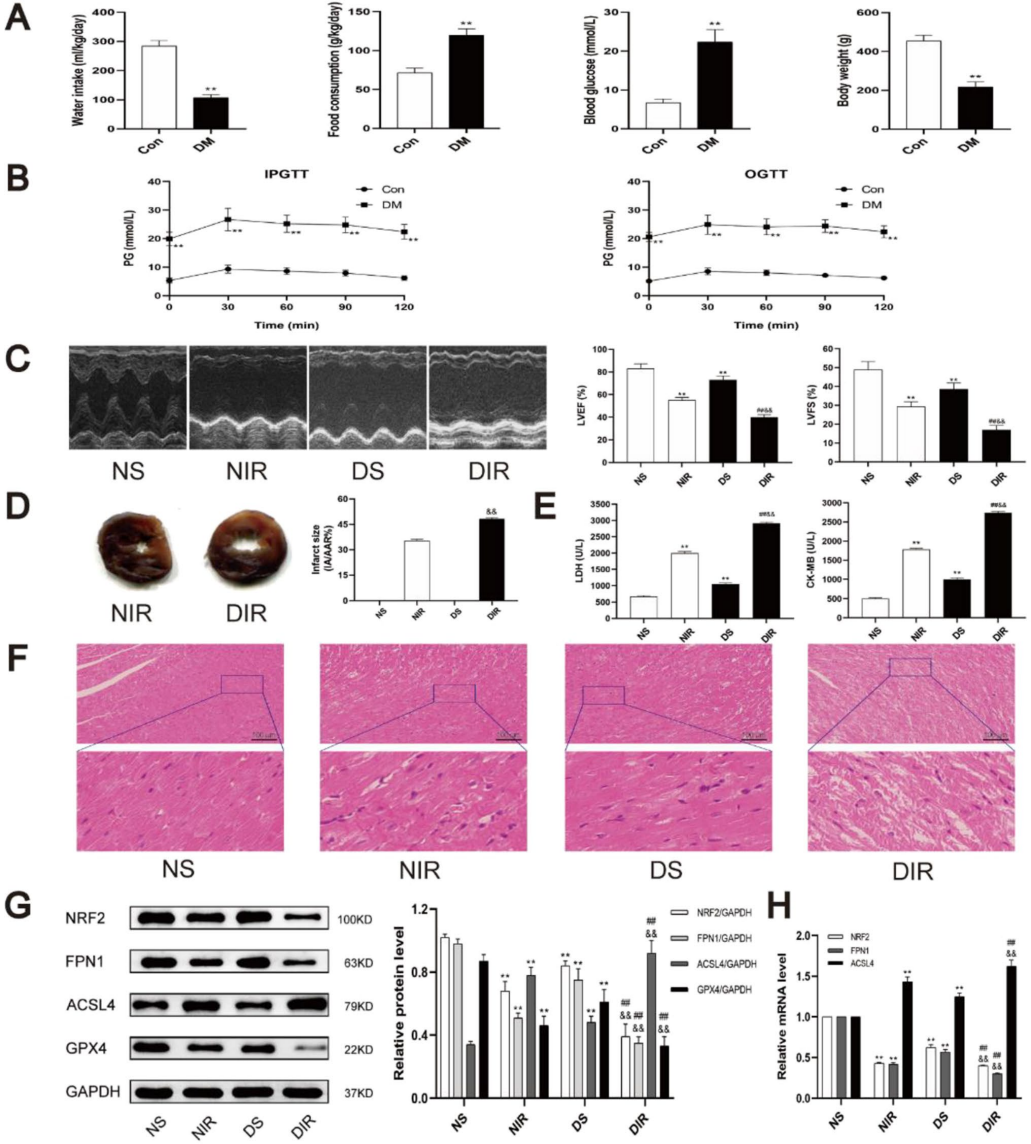
The effects of diabetes and myocardial IRI on ferroptosis and NRF2 pathway. **A** Basic characteristics of diabetic rats. Water intake and food consumption were the average value of 8 weeks. Body weight and plasma glucose were recorded before inducing myocardial ischemia–reperfusion. **B** Intraperitoneal glucose tolerance test and oral glucose tolerance test of diabetic rats. **C** Representative M-mode images by echocardiography. **D** Infarct size was detected by TTC. **E** The levels of CK-MB and LDH in serum were detected. **F** Histopathological changes of myocardium were detected by HE staining. **G** The protein levels of NRF2, FPN1, ACSL4, and GPX4 in myocardial tissue were detected by western blot. **H** The mRNA levels of NRF2, ACSL4, and GPX4 in myocardial tissue were detected by qRT-PCR. The results are expressed as the means ± SD. *N* = 8 for each group. ^**^P < 0.05 vs. NS group. ^##^*P* < 0.05 vs. NIR group. ^&&^*P* < 0.05 vs. DS group. *IPGTT* intraperitoneal glucose tolerance test, *OGTT* oral glucose tolerance test

After STZ-induced diabetes, the diabetic rats showed characteristic symptoms of diabetes including hyperglycemia, polydipsia, polyphagia, and weight reduction (Fig. 2A). Moreover, STZ-induced rats exhibited markedly impaired IPGTT and OGTT (Fig. 2B). Therefore, these data demonstrated that our diabetic animal models were successfully developed.

Ischemia–reperfusion significantly impaired cardiac function by decreasing LVEF and LVFS in both diabetic and non-diabetic animals. However, compared with the NIR group, DIR group showed further impaired cardiac function (Fig. 2C). Uniformly, compared with the NIR group, the diabetes markedly increased myocardial infarction area after ischemia–reperfusion treatment (Fig. 2D). In addition, compared with normal rats, diabetic rats had higher levels of LDH and CK-MB (Fig. 2E). HE staining showed that the structure of myocardial tissue in NS group was intact and the cardiomyocytes were arranged neatly. The myocardial tissue in DS and NIR groups showed mild disorder of cell arrangement and partial myocardial borders breakage. In the DIR group, the myocardial tissue showed severe cell alignment disorder, cell swelling, and necrosis, and most of the myocardial fibers were tremendously broken (Fig. 2F). The above results indicated that the myocardial IRI was significantly exacerbated in diabetic rats compared with non-diabetic rats.

Next, to determine the level of transcription in cardiomyocytes NRF2 and FPN1, we assessed the mRNA in cardiomyocytes in each group. Our experiments found that compared with the NS group, the protein and mRNA expression of NRF2 and FPN1 in DS and NIR group was lower (Fig. 2G–H). The mRNA and protein expression levels of NRF2 and FPN1 were significantly reduced in the DIR group. Western blot showed that compared with the NS group, the expression of ACSL4 in the DS group and the NIR group increased, and the expression of ACSL4 in the DIR group further increased (Fig. 2G). However, the level of GPX4 was opposite to ACSL4. And the expression level of mRNA of ACSL4 is consistent with protein (Fig. 2H). These data indicated that following myocardial ischemia–reperfusion treatment, impairment of the NRF2/FPN1 signaling pathway caused development of ferroptosis to exacerbate the myocardial IRI, in diabetic rats compared with that in non-diabetic rats.

### The ferroptosis inducer abrogated the protective effect of NRF2 activator SFN in diabetic rats following myocardial IRI

**Fig. 3:**
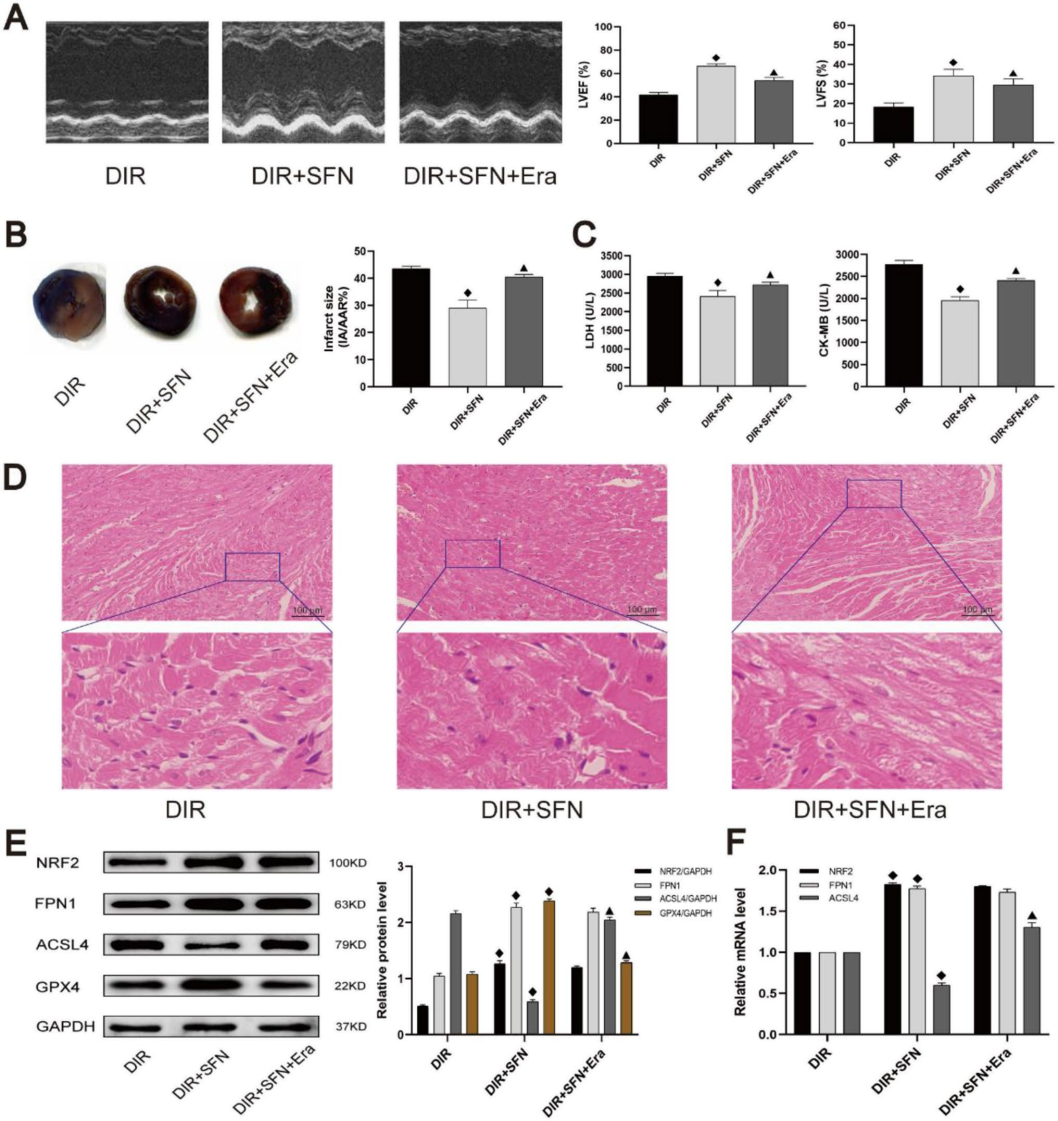
The ferroptosis inducer could abrogate the protective effect of activating NRF2 pathway in diabetic rats following myocardial IRI. **A** Representative M-mode images by echocardiography. **B** Infarct size was detected by TTC. **C** The levels of CK-MB and LDH in serum were detected. **D** Histopathological changes of myocardium were detected by HE staining. **E** The protein levels of NRF2, FPN1, ACSL4, and GPX4 in myocardial tissue were detected by western blot. F The mRNA levels of NRF2, ACSL4, and GPX4 in myocardial tissue were detected by qRT-PCR. The results are expressed as the means ± SD. *N* = 8 for each group. ^◆^*P* < 0.05 vs. DIR group. ^▲^*P* < 0.05 vs. DIR + SFN group. *SFN* sulforaphane, *Era* erastin

To verify the contribution of NRF2/FPN1-dependent ferroptosis to diabetic myocardial IRI, the NRF2 activator SFN was applied to activate the NRF2/FPN1 signaling pathway. We found that activation of NRF2 could distinctly alleviate myocardial IRI in diabetic rats. SFN significantly improved post-myocardial IRI cardiac functional recovery by increasing LVEF and LVFS, and the size of myocardial infarction was also reduced (*P* < 0.05). While the ferroptosis inducer Erastin attenuated cardioprotective effect of SFN (Fig. 3A and B), the LDH and CK-MB levels in the DIR + SFN group were lower compared with the DIR group (Fig. 3C), indicating that the degree of myocardial IRI was reduced after SFN administration. Through HE staining observation, compared with the DIR group, the left ventricular structural damage in the DIR + SFN group were reduced (Fig. 3D).

Western blot and qRT-PCR showed that after pretreatment with SFN, the expression of NRF2, FPN1, and GPX4 in rat myocardium were upregulated, the expression of ACSL4 was downregulated (Fig. 3E and F). When exposed to Erastin treatment, the above effects of SFN were all inhibited, and the expression of GPX4 was lower than that in DIR + SFN group, but the expression of ACSL4 was higher (Fig. 3A–F). These results collectively suggest that the protective effect on myocardial IRI by activating the NRF2/FPN1 signaling pathway was achieved through inhibition of ferroptosis.

### Downregulation of NRF2/FPN1 pathway was accompanied by ferroptosis in H9c2 cardiomyocytes exposed to HH/R

**Fig. 4:**
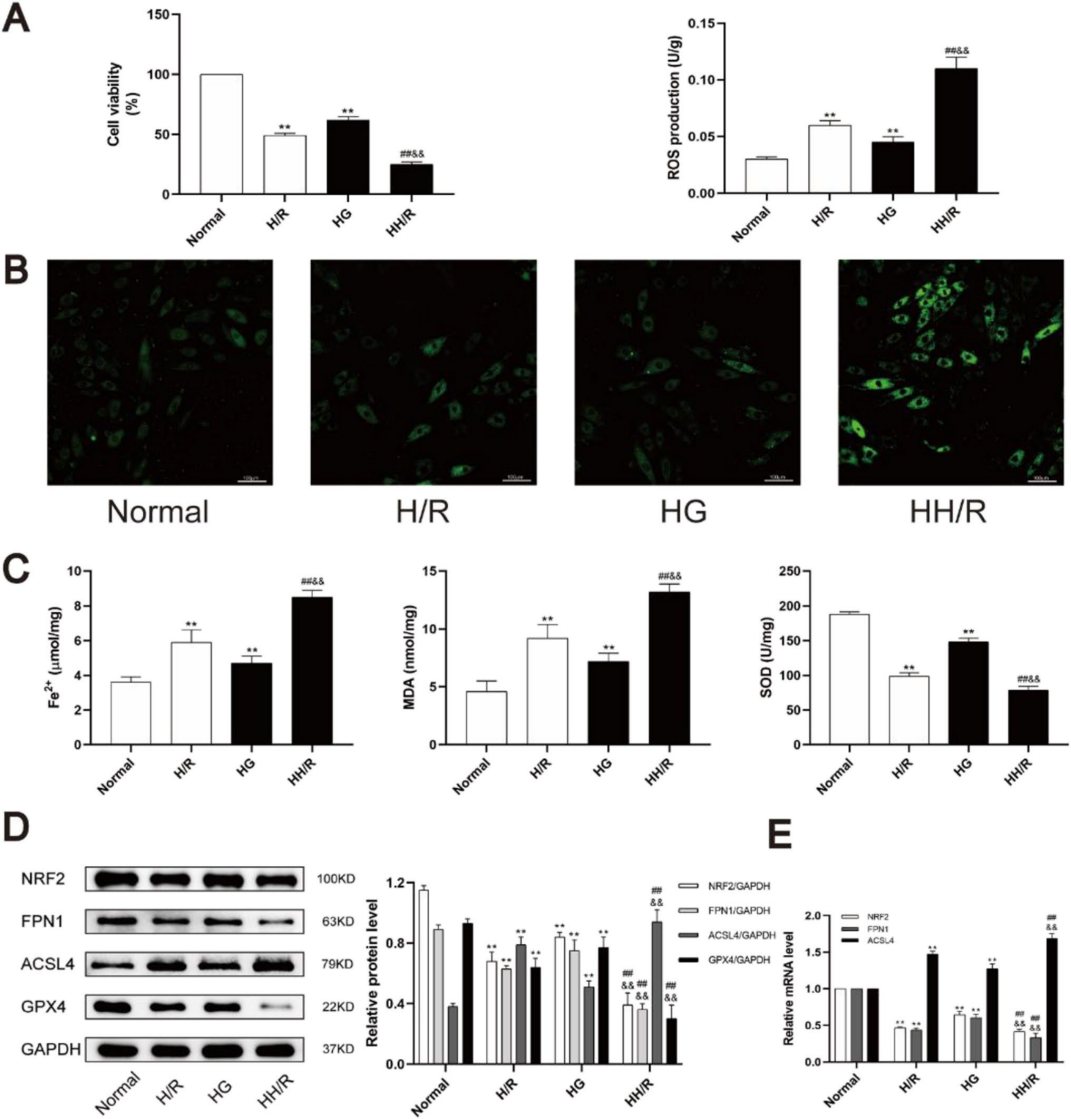
Downregulation of NRF2 pathway is accompanied by ferroptosis in H9c2 cardiomyocytes exposed to HH/R. **A** Cell viability was detected by CCK-8. **B** ROS production was measured by adding the fluorescent probes to the cells during 20 min at 37 ℃. Scale bars: 100 μm. **C** Fe^2+^ content, MDA, and SOD levels in H9c2 cells were detected. **D** The protein levels of NRF2, FPN1, ACSL4, and GPX4 in myocardial tissue were detected by western blot. **E** The mRNA levels of NRF2, ACSL4, and GPX4 in myocardial tissue were detected by qRT-PCR. ^**^P < 0.05 vs. NS group. ^##^*P* < 0.05 vs. NIR group. ^&&^*P* < 0.05 vs. DS group

In order to identify further molecular mechanism of NRF2/FPN1 pathway in diabetic myocardial IRI, H9c2 cardiomyocytes were used for in vitro experiments. Compared with normal group, the cell viability of H9c2 cells in HG group and H/R group were decreased, and lipid ROS production were increased (Fig. 4A and B). HG and H/R treatment increased intracellular Fe^2+^ and MDA content and decrease SOD activity (Fig. 4C). And the above changes were more obvious in the HH/R group (*P* < 0.05). The conditions were consistent with in vitro experiments, and HG and H/R treatment downregulated the expression of NRF2, FPN1, and GPX4 at the same time upregulated the expression of ACSL4 (Fig. 4D and E). From these findings, we could infer that hypo-expression of the NRF2/FPN1 signaling pathway in H9c2 cardiomyocytes exposed to HH/R could cause iron over-load and lipid peroxide accumulation, which in turn results in ferroptosis.

### The protective effects of NRF2 activator SFN in H9c2 cardiomyocytes from HH/R-induced ferroptosis were reversed by erastin

**Fig. 5:**
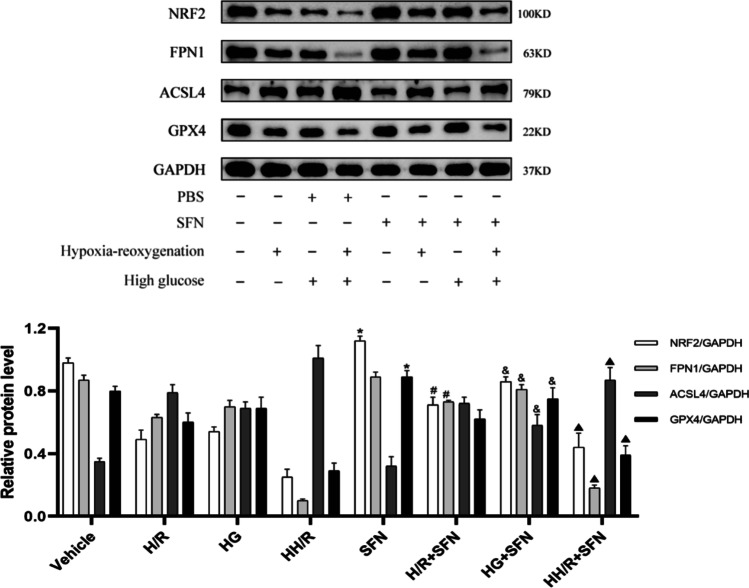
The effects of NRF2 activator SFN on HG and H/R-treated H9c2 cardiomyocytes. ^*^P < 0.05 vs. vehicle group. ^#^*P* < 0.05 vs. H/R group. ^&^*P* < 0.05 vs. HG group. ^▲^*P* < 0.05 vs. HH/R group. *SFN* sulforaphane

**Fig. 6:**
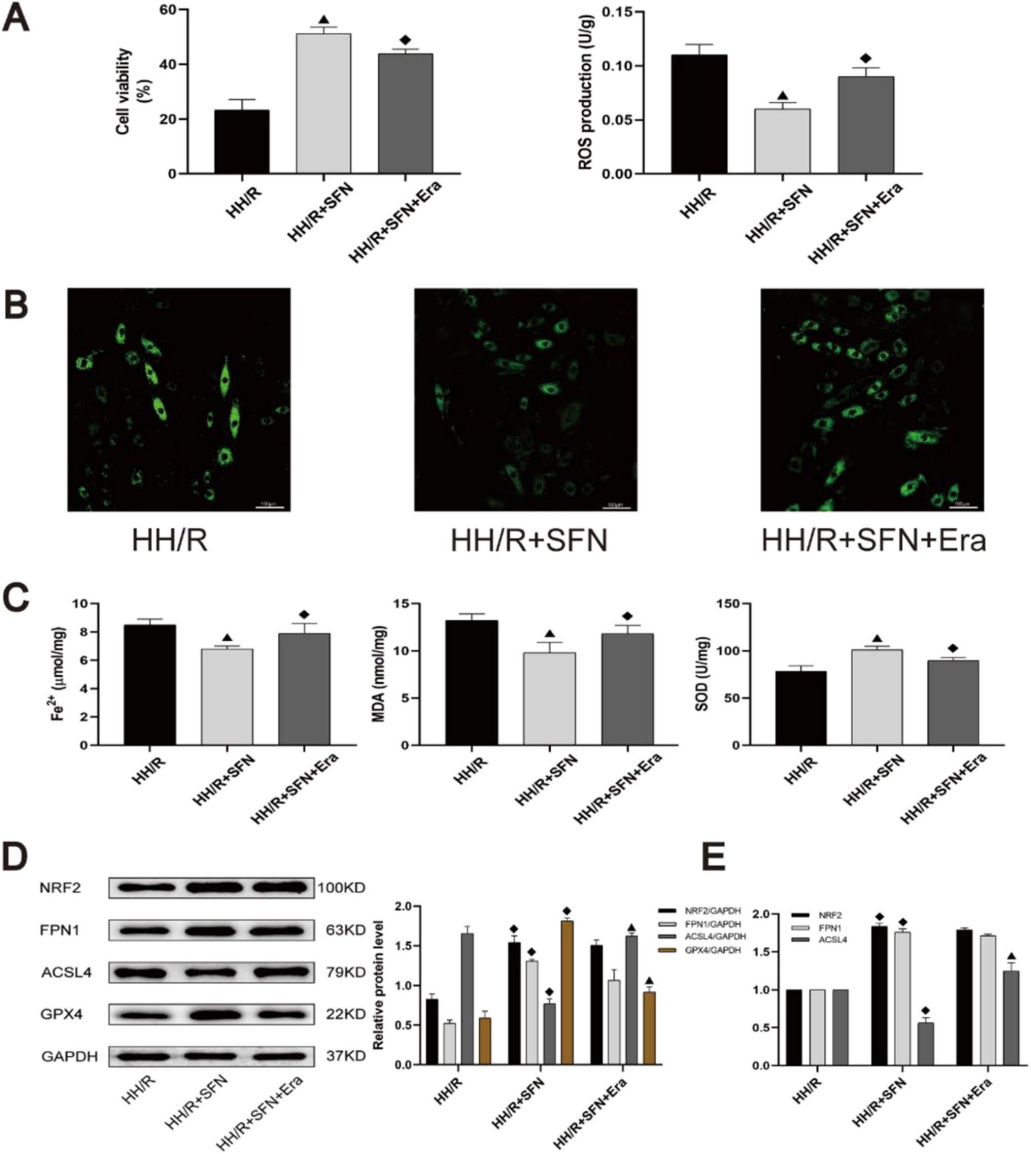
The beneficial effects of overexpression of NRF2 pathway to protect H9c2 cardiomyocytes from HH/R-induced ferroptosis were reversed by erastin. **A** Cell viability was detected by CCK-8. **B** ROS production was measured by adding the fluorescent probes to the cells during 20 min at 37 ℃. Scale bars: 100 μm. **C** Fe^2+^ content, MDA, and SOD levels in H9c2 cells were detected. **D** The protein levels of NRF2, FPN1, ACSL4, and GPX4 in myocardial tissue were detected by western blot. **E** The mRNA levels of NRF2, ACSL4, and GPX4 in myocardial tissue were detected by qRT-PCR. ^◆^*P* < 0.05 vs. DIR group. ^▲^*P* < 0.05 vs. DIR + SFN group. *SFN* sulforaphane, *Era* erastin

To identify the possible mechanisms underlying the protective effects of NRF2 in HH/R-induced ferroptosis, H9c2 cardiomyocytes overexpressing NRF2 were obtained via administration with SFN. As depicted in Fig. 5, compared to the other groups, the HH/R and SFN treatment group showed more significant upregulation of NRF2/FPN1 signaling pathway and more pronounced changes in ferroptosis-related proteins (*P* < 0.05). Therefore, in line with our expectations, we focused our study mainly on the HH/R group. As showed in Fig. 6A, the cell viability was increased in the HH/R + SFN group. Compared with HH/R group, the ROS fluorescence intensity was significantly reduced after SFN incubation (Fig. 6B). Besides, SFN treatment significantly attenuated intracellular Fe^2+^ content and lipid peroxide accumulation by impacting SOD and MDA levels (Fig. 6C). After NRF2 was activated by SFN, the expression of iron metabolism-related protein FPN1 was also increased accordingly, with reduced indicators of ferroptosis (Fig. 6D and E). Furthermore, as seen in Fig. 6A–E, after pretreatment with erastin, the expression of NRF2 and FPN1 did not change significantly with increased ferroptosis level (*P* > 0.05). Collectively, ferroptosis inducer reversed the beneficial effect of overexpression of NRF2/FPN1 signaling pathway to protect H9c2 cardiomyocytes from HH/R-induced ferroptosis.

### *NRF2 activator SFN protected H9c2 cardiomyocytes from HH/R-induced ferroptosis by transcribing downstream iron metabolism-related gene *via* nuclear translocation*

**Fig. 7:**
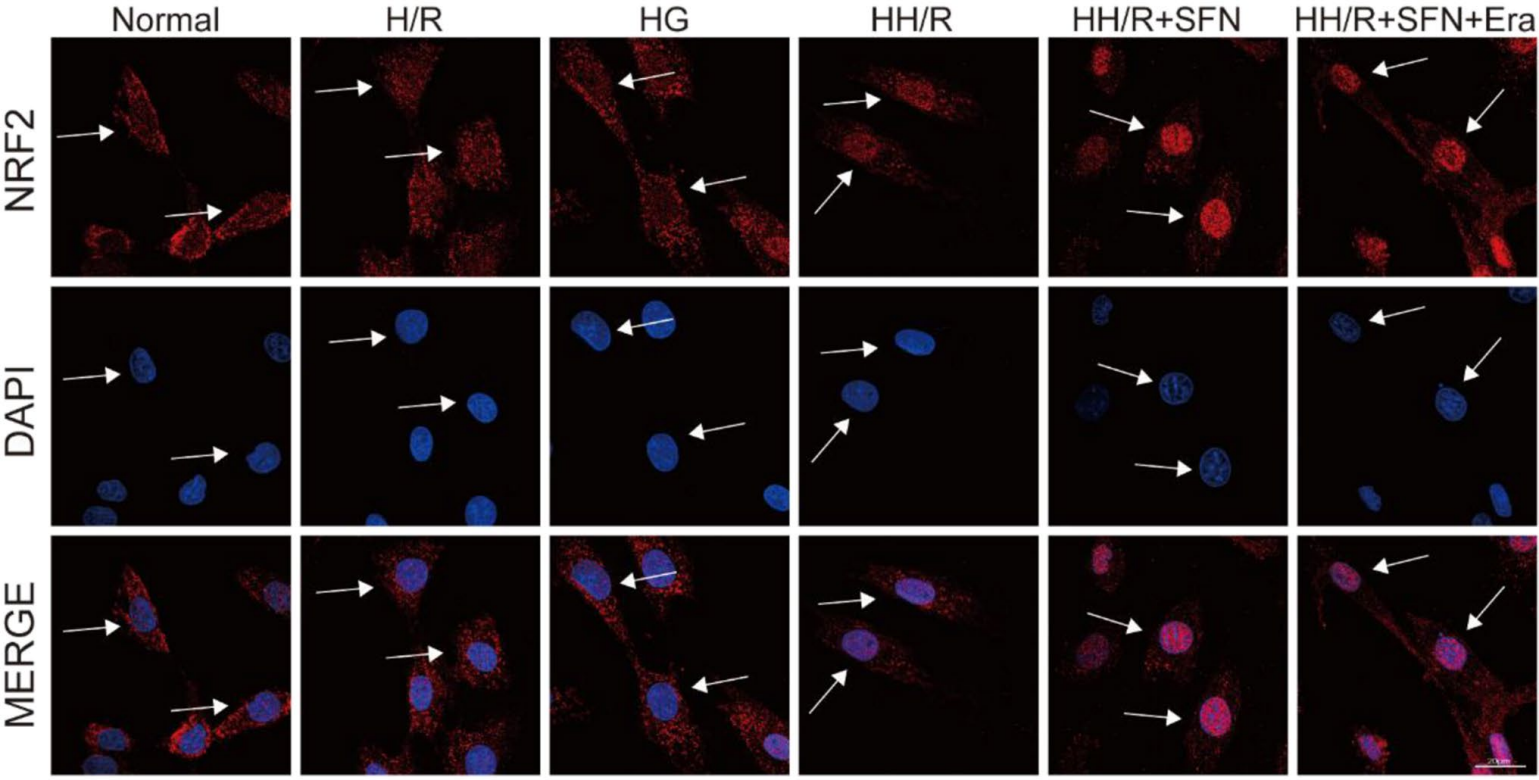
Immunofluorescence staining of NRF2 using H9c2 cardiomyocytes after corresponding stimulation. The red dots represent NRF2, and blue dots show nuclear. Scale bars: 20 μm. *SFN* sulforaphane, *Era* erastin

To visualize the nuclear localization of NRF2, an immunofluorescence assay was performed in H9c2 cardiomyocytes. As presented in Fig. 7, compared with normal group, there was a significant decrease in NRF2 protein expression in the nucleus relative to the cytoplasm following the treatment of HH/R (*P* < 0.05). Moreover, SFN pretreatment significantly increased the nuclear translocation of NRF2 (*P* < 0.05). Taken together, these data suggested that SFN protected H9c2 cardiomyocytes from ferroptosis damage by modulating NRF2 nuclear accumulation.

## Discussion

In this study, we attempted to understand whether ferroptosis, an iron-dependent regulated form of cell death caused by iron dysmetabolism and accumulation of lipid peroxides, may underlie the pathogenesis of myocardial IRI in diabetic rats. Furthermore, we first evaluated whether the activation of NRF2/FPN1 signal pathway could prevent ferroptosis in diabetic myocardial IRI. In addition, nuclear transfer of NRF2 plays a pivotal role in ferroptosis in STZ-induced diabetic IRI. To our knowledge, this is the first study to examine the mechanism of NRF2-mediated cardio-protection by regulating iron metabolic balance and iron homeostasis.

In the present study, we respectively established diabetes and ischemia–reperfusion models in rats to simulate myocardial ischemia–reperfusion in diabetic patients. For the in vivo experiments, it was obviously showed that diabetes could increase the vulnerability of the heart following ischemia–reperfusion. When the body’s tissues or organs are damaged, cellular metabolism gets affected and PH decreases, resulting in the reduction of intracellular Fe^3+^ to Fe^2+^, overloaded iron ions can form highly reactive and toxic hydroxyl radicals via the Haber–Weiss/Fenton reactions, which in turn can cause ferroptosis (Qi et al. 2020). Furthermore, related research has shown that supply of exogenous iron such as ferric ammonium citrate, ammonium citrate, and ferric citrate can enhance the ferroptosis induced by erastin and RSL3 (Manz et al. 2016), while the antioxidant vitamin E can inhibit ferroptosis (Kagan et al. 2017). It is thus clear that iron metabolic homeostasis and oxidative stress play key roles in the pathogenesis of ferroptosis. The final product of membrane lipid peroxidation reaction, MDA, might reflect the degree of lipid peroxidation reaction beginning. SOD, which is commonly found in aerobic organisms, is an important member of the antioxidant enzyme class and is one of the main enzymes for effective scavenging of ROS in organisms. By measuring the content of Fe^2+^, SOD, and MDA in myocardial tissues, our study found iron ions accumulation and imbalance of oxidative and antioxidant systems in rats’ hearts under diabetes and myocardial IR conditions. We therefore hypothesized that ferroptosis may have occurred during diabetic myocardial IRI. Acyl-CoA synthetase long-chain family member 4 (ACSL4) is a reliable marker of ferroptosis and induces the production of the signature signal of ferroptosis, 5-hydroxyeicosatetraenoic acid (5-HETE) (Yuan et al. 2016). GPX4 is the only enzyme in the GPX family that reduces esterification of oxidized fatty acids and cholesterol hydroperoxides (Doll et al. 2019). As we expected, the mRNA and protein expression of ACSL4 and GPX4 were changed during diabetic myocardial IRI, which was in line with previous reports (Wang et al. 2020; Zhao et al. 2017). Notably, our previous study also confirmed the fact that ferroptosis is involved in regulating myocardial IRI in diabetic rats, and these were consistent with our present results (Li et al. 2020, 2021). From these, we conclude that the increased vulnerability of myocardial IRI in diabetic patients is strongly linked to the disorders of iron metabolism and ferroptosis.

A growing number of experimental studies have illustrated that NRF2 can attenuate diabetic myocardial IRI and effectuate cardioprotective effects (Zhou et al. 2021). In present study, our results showed that the level of NRF2 was downregulated after IR induction as well as during hyperglycemic states, which was consistent with the conclusion of Liu et al. (Liu et al. 2020b) and Tan et al. (Tan et al. 2011). Therefore, we inferred that the downregulation of NRF2 expression in the diabetic heart exacerbates oxidative stress and insulin resistance, resulting in exacerbation of myocardial damage. SFN is a well-studied classic NRF2 activator that prevents oxidative stress injury and the accompanying cardiovascular disease. In consideration of the fact that the pathogenesis of diabetic cardiomyopathy is a complex chronic process, fast drug administration of SFN has limited cardioprotection in diabetic rats. Consequently, we referred to Piao et al. who chose to inject SFN (500 μg/kg/day) intraperitoneally for 3 consecutive days before ischemia (Piao et al. 2010). SFN administration dramatically reduced diabetic myocardial IRI, as shown by smaller infarcts, lower serum biomarker levels, and preserved cardiac function, according to our findings. The NRF2 has been widely reported to play a novel role in diabetic myocardial IRI and also participates in ferroptosis (Fang et al. 2019; Xiao et al. 2019). Meanwhile, some studies proved that at the transcriptional level, NRF2 could regulate the expression of FPN1, a protein that exerts an important role in iron homeostasis at the systemic level and is currently the only known iron release related protein in mammals (Donovan et al. 2005; Marro et al. 2010). Additionally, our data suggested that compared with the DIR group, NRF2, FPN1, and GPX4 expressions were upregulated, ACSL4 expression was downregulated, myocardial Fe^2 +^ and MDA content was decreased, and SOD activity was increased in the DIR + SFN group, implying that SFN can reduce myocardial IRI in diabetic rats by promoting FPN1-mediated Fe^2+^ efflux and in turn limiting ferroptosis.

**Fig. 8:**
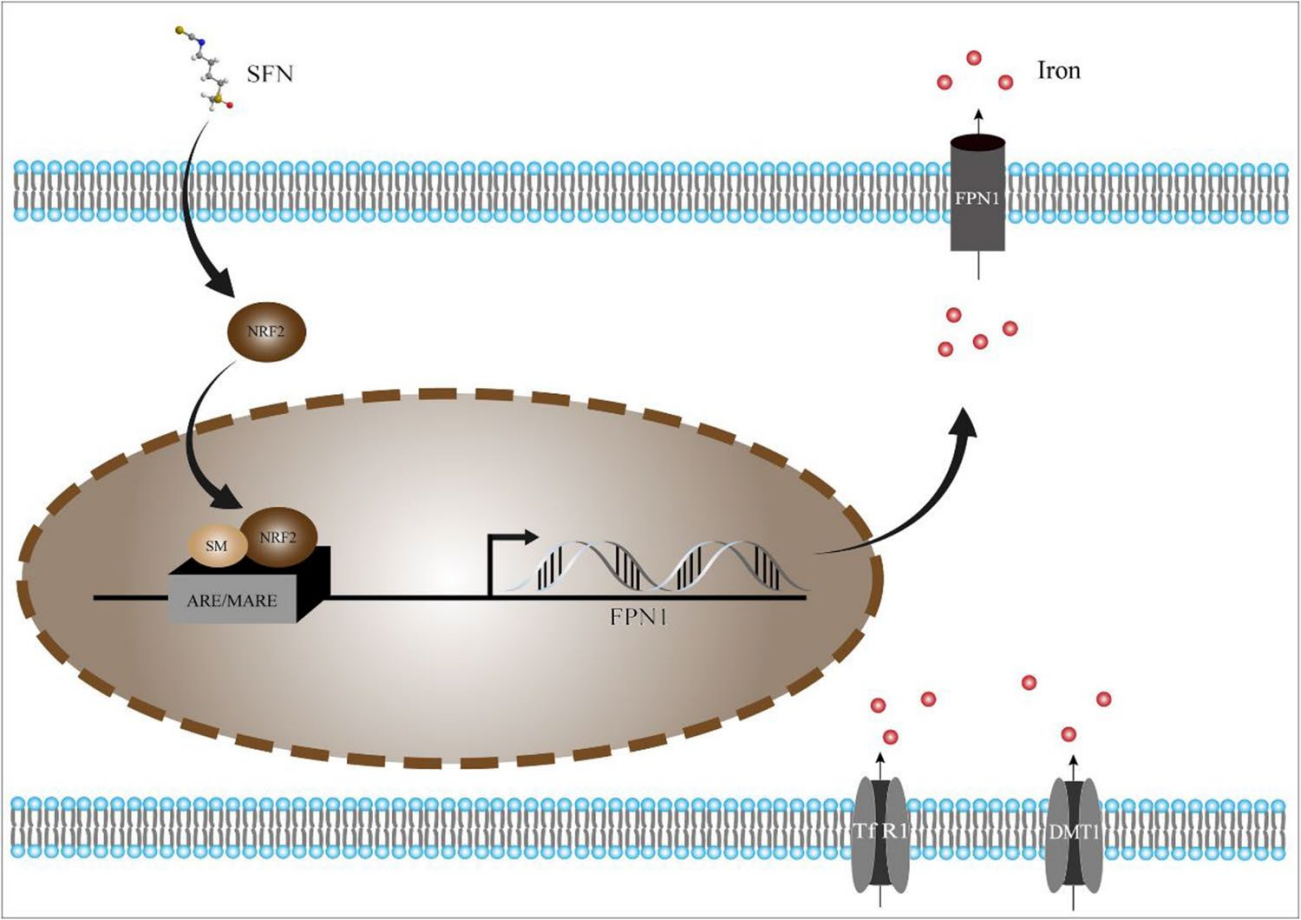
The effects of NRF2 activation on iron metabolism in cardiomyocytes. After SFN pretreatment, activated NRF2 translocates from the cytoplasm to the nucleus. Subsequently, NRF2 forming a heterodimer with small MAFs (SM) enhances the transcription of FPN1. Overloaded free iron is exported to the plasma via FPN1, which proves the protective effect of the NRF2 induction against ferroptosis. *SFN* sulforaphane

To further test our hypothesis, the specific mechanism was verified in H9c2 cells in detail. There have been a lot of studies showed the involvement of oxidative stress in diabetes as well as in myocardial IRI (Zhao et al. 2017). ROS has also been shown to cause cell necrosis and tissue damage, either directly or indirectly (Ma 2010). H9c2 cells induced by HG or H/R were subjected to severe damage, and their ROS levels were elevated, which was consistent with in vivo experimental data. However, the mRNA and protein expression of NRF2 and FPN1 were decreased. This was consistent with the findings of Xu et al. (Xu et al. 2019). We consider that it may be due to the fact that diabetic heart disease is a chronic disease model where long-term oxidative stress depletes antioxidant elements such as NRF2. In further study, the application of SFN rescued this situation and thereby alleviated HH/R damage in H9c2 cells. Notably, it has been recently shown that changing NRF2 expression in the nucleus can impact its transcriptional target FPN1, resulting in decreased iron efflux and increased intracellular iron content (Yang et al. 2019). Similarly, in our study, our cellular immunofluorescent analysis revealed that NRF2 translocates from the cytoplasm to the nucleus in the process of H9c2 cells damage induced by HG or H/R, thereby activating the transcription of its target gene FPN1 to limit ferroptosis. Overall, in response to oxidative stress, we speculated that NRF2 was released from the cytoplasm and translocated into the nucleus, where it bound to the upstream promoter region of the antioxidant response element (ARE) and members of the sMaf protein family. As shown in Fig. 8, the above process eventually resulted in transcription of downstream protective protein genes like FPN1 and heightened cellular resistance to ferroptosis.

Erastin, a ferroptosis inducer that acts on the cystine/glutamate antiporter system Xc-, is the most widely utilized ferroptosis inducer today (Yang et al. 2020). Surprisingly, the administration of erastin prevented the positive effects of SFN and increased the degree of ferroptosis while having no effect on the expression of NRF2 and FPN1, indicating that ferroptosis is controlled by targeting the NRF2/FPN1 signaling pathway. Together, we can conclude that hypo-expression of FPN1 can lead to intracellular iron accumulation and aggravate ferroptosis in the diabetic heart disease, and that SFN further stimulates NRF2 to enhance the transcription of FPN1 and reduce myocardial IRI in diabetic rats.

This study has certain drawbacks. On the one hand, our study is limited to STZ-induced type 1 diabetic rats. Because type 2 diabetes is the most frequent type of diabetes, determining the importance of the NRF2/FPN1 signaling pathway in this illness is crucial. On the other hand, due to the experiment’s technical constraints, we were unable to successfully extract NRF2 from the nucleus of the cells. Despite the fact that NRF2’s transcriptional action is performed in the nucleus, no research have clearly demonstrated the degree of NRF2 translocation from the cytoplasm to the nucleus, which was one of the key reasons why we decided to extract the total NRF2. Precisely for these reasons, further study is still needed to uncover the underlying molecular mechanism.

In conclusion, our present study demonstrated that NRF2/FPN1 signal pathway is a pivotal mechanism in diabetic myocardial IRI by regulating iron metabolic homeostasis to restrict ferroptosis, and activation of NRF2/FPN1 signal pathway can relieve diabetic myocardial IRI to some extent. Modulating ferroptosis by impacting iron metabolism might provide an effective strategy for the prophylaxis and treatment of diabetic myocardial IRI.

## Abbreviations

AAR: Area at risk
ACSL4: Acyl-CoA synthetase long-chain family member 4
CCK-8: Cell counting kit-8
CK-MB: Creatine kinase-MB
DAPI: 4′,6-Diamidino-2-phenylindole dihydrochloride
DCFH-DA: 2′,7′-Dichlorofluorescein diacetate
DMEM: Dulbecco’s modified Eagle’s medium
DIR: Diabetes and ischemia–reperfusion
DS: Diabetes and sham
ECG: Electrocardiogram
Era: Erastin
FBS: Fetal bovine serum
FPN1: Ferroportin1
GPX4: Glutathione peroxidase 4
HE: Hematoxylin and eosin
HH/R: High glucose and hypoxia-reoxygenation
HG: High glucose group
H/R: Hypoxia-reoxygenation group
IA: Infarct area
IPGTT: Intraperitoneal glucose tolerance test
IRI: Ischemia-reperfusion injury
LAD: Left anterior descending coronary artery
LDH: Lactate dehydrogenase
LVEF: Left ventricular ejection fraction
LVFS: Left ventricular shortening fraction
MDA: Malondialdehyde
NIR: Normal and ischemia–reperfusion
NRF2: Nuclear factor E2-related factor 2
NS: Normal and sham
OGTT: Oral glucose tolerance test
ROS: Reactive oxygen species
SFN: Sulforaphane
SOD: Superoxide dismutase
STZ: Streptozotocin
TTC: Triphenyl tetrazolium chloride
